# Results of the first randomized controlled trial of integrated cognitive-behavioral therapy for eating disorders and posttraumatic stress disorder

**DOI:** 10.1017/S0033291721004967

**Published:** 2022-02

**Authors:** Kathryn Trottier, Candice M. Monson, Stephen A. Wonderlich, Ross D. Crosby

**Affiliations:** 1 Centre for Mental Health, University Health Network, Toronto, Canada; 2 Department of Psychiatry, University of Toronto, Toronto, Canada; 3 Department of Psychology, Ryerson University, Toronto, Canada; 4 Sanford Health, Fargo, North Dakota, USA; 5 Sanford Research, Fargo, North Dakota, USA; 6 Department of Psychiatry and Behavioral Science, University of North Dakota School of Medicine and Health Sciences, Grand Forks, North Dakota, USA

**Keywords:** Cognitive processing therapy, cognitive-behavioral therapy, eating disorders, integrated treatment, posttraumatic stress disorder

## Abstract

**Background:**

Eating disorders (EDs) and posttraumatic stress disorder (PTSD) frequently co-occur and can share a functional relationship. The primary aim of this initial randomized controlled trial was to determine whether integrated cognitive-behavioral therapy (CBT) for co-occurring ED-PTSD was superior to standard CBT for ED in improving PTSD symptoms. Intervention safety and desirability, as well as the relative efficacy of the treatments in improving anxiety, depression, and ED symptomatology, were also examined.

**Methods:**

Following a course of intensive ED treatment, individuals with ED-PTSD were recruited to participate and randomized to integrated CBT for ED-PTSD or standard CBT for ED. The sample consisted of 42 individuals with a range of ED diagnoses. Outcomes were assessed at end-of-treatment, 3-, and 6-month follow-up using interview and self-report measures.

**Results:**

Mixed models revealed significant interactions of time and therapy condition on clinician-rated and self-reported PTSD symptom severity favoring Integrated CBT for ED-PTSD. Both treatments were associated with statistically significant improvements in PTSD, anxiety, and depression. Improvements were maintained at 3- and 6-month follow-up. There was good safety with both interventions, and satisfaction with both treatments was high. However, there was a stronger preference for integrated treatment.

**Conclusions:**

Integrating CBTs for PTSD and ED following intensive ED treatment is safe, desirable, and efficacious for improving PTSD symptoms. Future studies with larger sample sizes are needed to determine whether Integrated CBT for ED-PTSD provides benefits over standard CBT for ED with respect to ED outcomes.

Posttraumatic stress disorder (PTSD) frequently co-occurs with eating disorders (EDs) in the general population (Dansky, Brewerton, Kilpatrick, & O'Neil, 1997; Hudson, Hiripi, Pope, & Kessler, 2007) and in treatment-seeking samples, with the highest rates observed in *intensive* ED treatment settings (inpatient, residential, partial hospitalization; e.g. Brewerton *et al*. 2020; Mitchell, Singh, Hardin, & Thompson-Brenner, 2021; Vanzhula, Calebs, Fewell, & Levinson, 2019). Although psychiatric comorbidity is common in both PTSD and EDs (Hudson et al., 2007; Kessler, Sonnega, Bromet, Hughes, & Nelson, 1995), co-occurring ED and PTSD (i.e. ED-PTSD) is of particular concern because of the potential for a functional relationship between the disorders that may increase the likelihood that the disorders maintain one another (Karr et al., 2013; Liebman et al., 2020; Vanzhula et al., 2019), and result in high rates of relapse following standard ED treatment (Berends, Boonstra, & van Elburg, 2018; Yu, Agras, & Bryson, 2013). ED-PTSD is also associated with greater ED severity (Rijkers, Maartje, van Hoeken, & Hoek, 2019), and other psychopathology (Brewerton et al., 2020; Scharff, Oritz, Forrest, & Smith, 2019; Trottier, 2020). Emerging research suggests that individuals with co-occurring PTSD may be more likely to end ED treatment early (Trottier, 2020), have poorer end-of-treatment (EoT) ED outcomes (Hazzard et al., 2021; Serra et al., 2020), and relapse following ED treatment (Scharff, Oritz, Forrest, Smith, & Boswell, 2021). Consistent with these findings, many frontline ED clinicians perceive trauma-related symptoms as a major obstacle to successful ED treatment and believe addressing trauma-related symptoms in individuals with EDs is important (Trottier, Monson, Wonderlich, MacDonald, & Olmsted, 2017).

Specific trauma-focused cognitive-behavioral therapies (CBTs) are the first-line treatments for PTSD (e.g. American Psychological Association, 2017). However, individuals with ED-PTSD are not often offered these treatments, in part, due to concerns that they are not ready for trauma-focused therapy, and that it may lead to worsening of ED, trauma-related, and other mental health symptoms (Trottier et al., 2017). Despite these perceived challenges, there have been repeated calls for the development and evaluation of integrated CBTs for ED-PTSD (e.g. Brewerton, 2007; Holzer, Uppala, Wonderlich, Crosby, & Simonich, 2008; Rijkers *et al*. 2019) and many clinicians believe concurrent approaches are most appropriate (Trottier et al., 2017).

To date, there is one published study of integrated psychotherapy for ED-PTSD. Trottier and colleagues developed integrated CBT for ED-PTSD by integrating cognitive processing therapy (CPT) for PTSD including a written trauma account (Resick, Monson, & Chard, 2016) with interventions from enhanced CBT for ED (CBT-E; Fairburn, Cooper, & O'Connor, 2008) – two leading evidence-based CBTs. In an uncontrolled study, integrated CBT for ED-PTSD was provided to 10 women following a course of intensive hospital-based ED treatment focused on nutritional stabilization and behavioral symptom interruption (Trottier et al., 2017). There were statistically significant and large magnitude improvements in PTSD symptoms from baseline to EoT, and 90% remitted from their PTSD diagnosis. There were no statistically significant changes in ED psychopathology over the course of integrated CBT, and 89% of participants who were remitted from their ED at study baseline (end-of-intensive ED treatment) remained remitted from their ED at the end of integrated CBT.

The current study is the first randomized controlled trial (RCT) examining the efficacy of integrated CBT for ED-PTSD. Our primary aim was to determine whether integrated CBT for ED-PTSD was superior to standard CBT for ED in improving PTSD symptoms. We also aimed to investigate the safety and desirability of integrated CBT for ED-PTSD. We hypothesized that integrated CBT would result in greater improvements in clinician-rated PTSD symptoms at EoT and follow-up. Secondary hypotheses were that integrated CBT would also result in greater improvements in self-reported PTSD symptoms, anxiety, and depression at EoT and follow-up. Additionally, we aimed to explore the impact of integrated CBT relative to standard CBT for ED on ED outcomes at EoT and follow-up.

## Method

### Design

The study was a parallel groups RCT with two active treatment arms. The trial involved a hybrid efficacy–effectiveness design in which the efficacy of integrated CBT for ED-PTSD was tested in a routine ED clinical practice setting with ED program therapists and patients. Following a course of intensive ED treatment, participants were randomly assigned to either (1) standard CBT for ED or (2) integrated CBT for ED-PTSD. The number and length of sessions were standardized across treatments. Participants were assessed at study baseline, end-of-study treatment (EoT), as well as 3 and 6 months after study treatment. Assessors were independent, and blind to treatment conditions. An intent-to-treat approach to data collection and analysis was utilized.

### Participants

Forty-three participants with ED-PTSD who received a course of day hospital or inpatient treatment for ED were randomized. However, one individual disclosed after randomization and before starting treatment that she had misrepresented her symptoms during the eligibility assessment and did not actually meet inclusion criteria. Thus, the individual was withdrawn and excluded resulting in a sample size of 42. Inclusion criteria were: Diagnostic and Statistical Manual of Mental Disorders, 5th Edition criteria (DSM-5; American Psychiatric Association, 2013) diagnosis of an ED at admission to intensive treatment, DSM-5 diagnosis of PTSD at study baseline, a body mass index (BMI; weight in kilograms/height in meters^2^) ⩾ 18.5, and stable or no psychotropic medication at study baseline. Diagnoses were assessed using evidence-based semi-structured interviews (described below). Exclusion criteria included medical conditions known to influence eating and/or weight, current substance dependence, uncontrolled bipolar or psychotic disorder, current participation in another psychosocial intervention or treatment study for ED or PTSD, and previous CPT.

### Measures

The Clinician-Administered PTSD Scale-5 (CAPS-5; Weathers et al., 2013) was used to make a PTSD diagnosis, and to determine PTSD remission. The CAPS-5 is a semi-structured clinician interview that assesses the severity of PTSD symptoms consistent with DSM-5 criteria (DSM-5; American Psychiatric Association, 2013). PTSD diagnostic status was based on meeting the DSM-5 symptom cluster criteria (to be counted a symptom, an item must be rated a 2 or higher). The past month CAPS-5 total severity score was the primary outcome measure. The range of scores on the CAPS-5 is 0–80, with higher scores indicating higher symptom severity. Internal consistency in the current study was adequate (*α* = 0.78).

The Eating Disorder Examination 17.0 (EDE; Fairburn, Cooper, and O'Connor, 2014) was used to assess ED diagnosis, remission, and severity of ED psychopathology. The EDE is a semi-structured clinician interview that assesses the frequency of ED behaviors and severity of ED psychopathology over the past 3 months. The past month EDE global score (measuring ED psychopathology) is a transdiagnostic measure of ED outcome, and is utilized in studies with mixed ED diagnosis samples (e.g. Fairburn et al., 2009). EDE global scores can range from 0 to 6, with higher scores reflecting greater psychopathology. Internal consistency in the current study was good (*α* = 0.86). ED diagnostic status also served as an ED outcome. There is currently no well-accepted definition of ED remission in the literature. Given the evidence that residual symptoms are common in remitted ED individuals (Tomba, Tecuta, Crocetti, Squarcio, & Tomei, 2019), we defined ED remission as no more than two objective binge and/or purge episodes in the past month and a BMI of greater than 18.5 which is in the range of previous definitions used in the literature (Tomba et al., 2019).

The Mini-International Neuropsychiatric Interview 7.0 (MINI-7; Sheehan, 2015) was used to assess exclusion criteria related to other mental health symptoms. The MINI-7 is a structured clinician-administered diagnostic interview assessing the presence of DSM-5 disorders in a number of categories, including mood disorders, anxiety disorders, obsessive-compulsive disorders, and substance use disorders.

The PTSD Checklist-5 (PCL-5; Blevins, Weathers, Davis, Witte, and Domino, 2015) served as a secondary measure of PTSD symptom severity. The PCL-5 is a self-report measure of PTSD symptoms over the past month corresponding with DSM-5 criteria. Scores on the PCL-5 range from 0 to 80, with higher scores indicating greater PTSD symptom severity. Participants also completed the Depression Anxiety Stress Scales (DASS; Lovibond and Lovibond, 1995) which provided measures of depression (score range, 0–42) and anxiety (0–42) over the past week with higher scores reflecting greater severity. All self-report measures had good internal consistency, *α* = 0.86 for the PCL-5, *α* = 0.91 for DASS depression, and *α* = 0.89 for DASS anxiety.

Treatment desirability was assessed at EoT via an investigator-constructed questionnaire. All participants rated their satisfaction with the treatment, and participants who received integrated CBT also rated the extent to which they believed having received integrated treatment would facilitate their ED recovery using a 5-point Likert Scale ranging from not at all to completely. All participants were also asked whether they would prefer integrated treatment for their ED and PTSD or separate treatments addressing one disorder at a time, as well as the extent to which they would recommend the treatment they received to someone else.

### Procedure

The first author's institutional review board approved the study protocol and all participants provided their written informed consent before eligibility assessment. Participants were recruited from the Eating Disorders Program at University Health Network, Toronto, Ontario, Canada between October 2015 and November 2018. Potential participants were identified by chart review and were provided with an overview of the study. Interested individuals then met with a member of the study staff to learn more about the study and for the informed consent process. Potential participants then underwent the eligibility/baseline assessment and eligible participants were randomized. Balanced block randomization was stratified according to ED diagnosis (anorexia nervosa *v.* other ED diagnosis). The study biostatistician generated the randomization, and allocation was concealed in envelopes that were opened by the study research assistant together with the participant when they were deemed eligible. Independent master's or doctoral-level clinicians conducted the CAPS-5, EDE, and MINI-7 interviews.

### Treatments and treatment fidelity

Both treatments consisted of 16 individual therapy sessions delivered over 14 weeks. Sessions 1–8 were delivered at a frequency of twice per week, sessions 9–12 were delivered on a weekly basis, and sessions 13–14 were delivered at 2-week intervals. Sessions 1–3 and 15–16 were 50 min and sessions 4–14 were 90 min in length.

CBT for ED was manualized by the first author by adapting interventions from CBT-E (Fairburn et al., 2008) for delivery following intensive ED treatment. Consistent with the hybrid efficacy-effectiveness trial design, the comparison condition (i.e. CBT for ED) was modeled after the treatment that was offered as part of routine care in the clinical program where the study was conducted. As in the prior pilot study, integrated CBT for ED-PTSD was a manualized treatment consisting of a combination of interventions from CPT (Resick et al., 2016) and CBT-E. Details on the content of the treatment are described by Trottier and Monson (2021). The intensive ED treatment that preceded the study treatments was focused on interrupting binge eating and purging, establishing a pattern of regular, nonrestrictive eating, and weight restoration (as relevant).

Four psychologists from the University Health Network ED program provided both of the study treatments. All therapists had been previously trained in CPT and CBT-Enhanced including attendance at in-person clinical training delivered by developers of the treatments. In order to promote adherence to the treatment protocols, therapists participated in a weekly group case consultation with the first and/or second author focused on the application of the treatments in accordance with study protocols. Therapy sessions were audio-recorded for consultation purposes and for fidelity assessment. Ten percent of therapy sessions in each condition were rated for manual adherence and therapist competence by four independent raters trained to reliability. An established CPT fidelity rating scale (Wiltsey Stirman, Monson, & Resick, 2017) was used to rate competence (range 0–6, with 0 indicating no evidence of competence and 6 indicating exceptional competence) and adherence (range 0–1, with 0 indicating a lack of adherence and 1 indicating adherence) with respect to the CPT components of integrated CBT for ED-PTSD. The same rating scale was adapted to rate adherence and competence with respect to the CBT for ED interventions in both treatment conditions. A score of 3 or higher on the competence rating scale indicated adequate competence. Adherence was very strong, with 100% of essential elements delivered in the CBT for ED condition, and 98.6% delivered in the integrated CBT for ED-PTSD condition. One hundred percent of sessions in the CBT for ED condition, and 85% of sessions in the CBT for ED-PTSD condition had a mean competence rating over 3.

### Statistical analyses

Power analysis was conducted to ensure adequate power to detect between-groups differences in clinician-rated PTSD at EoT and follow-up. The sample size was calculated assuming the use of multilevel modeling, an effect size of *g* = 0.80 based on prior studies of CBTs for PTSD (Watts et al., 2013), a conservative estimated correlation between repeated administrations of the CAPS-5 of *r* = 0.65, estimated measurement attrition of 20%, and a 2-tailed test with alpha equal to 0.05. A sample size of 40 participants was estimated to yield power greater than 90% to find the expected effect.

Analyses were conducted following intent-to-treat principles. All available data at each time-point were used in the multilevel models conducted using SPSS software version 25. Multilevel modeling was conducted on each outcome with a random intercept, and fixed effects for condition, time, and the condition × time interaction, and a first-order autoregressive covariance structure to account for the association between repeated measures. The primary outcomes were mean clinician-rated PTSD symptoms (CAPS-5 scores) at EoT and follow-ups compared between treatment conditions. The secondary and exploratory outcomes (anxiety, depression, and ED psychopathology) were analyzed using the same method. Pairwise comparisons were examined if omnibus tests were significant. To examine change over the course of treatment and maintenance of treatment gains, pairwise comparisons were conducted comparing baseline scores with EoT scores, and EoT scores with scores at 3- and 6-month follow-up. We also examined reliable change (Jacobson & Truax, 1991) in CAPS-5 and EDE global scores, as well as loss of diagnostic status (PTSD and ED) at each assessment point.

## Results

### Participant characteristics and flow

**Fig. 1.**
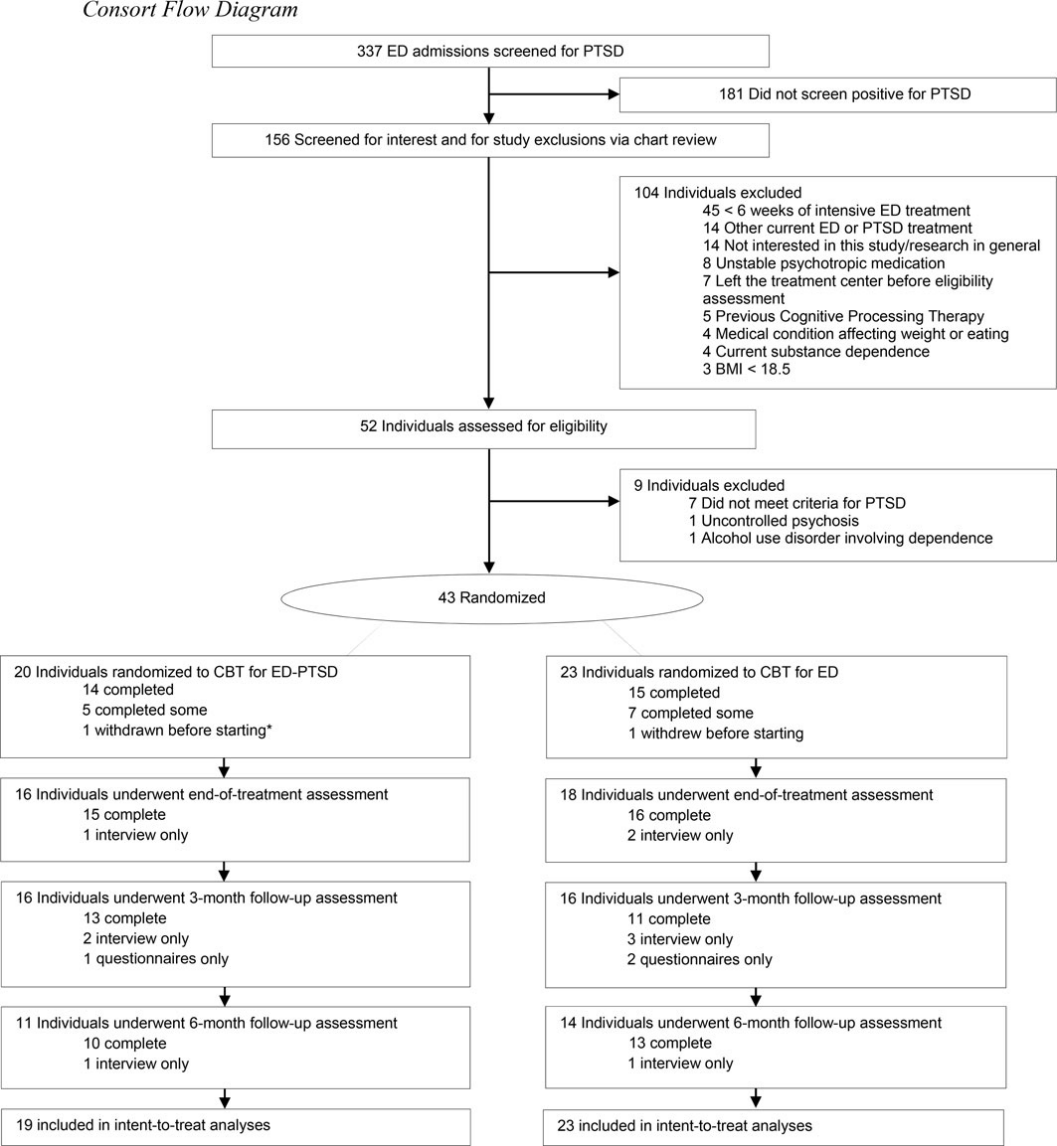
Consort flow diagram. *Note.* ED, eating disorder; PTSD, posttraumatic stress disorder; BMI, body mass index; CBT, cognitive-behavioral therapy. *Participant disclosed after randomization that they misrepresented their symptoms in the eligibility assessment and was subsequently deemed ineligible and excluded from analyses.

Table 1 shows the characteristics of the sample within each therapy condition at study baseline, and Fig. 1 shows the flow of participants through the study. There was no statistical difference in non-completion rate by condition [integrated CBT for ED-PTSD, *n* = 5 (26%); CBT for ED only, *n* = 8 (35%), Fisher's Exact *p* = 0.74). One participant withdrew before starting the study therapy because they were unwilling to accept randomization to the CBT for ED only condition. One participant was withdrawn by the study team due to repeated treatment interfering behaviors on hospital property which prevented appropriate engagement with treatment. There were no participants withdrawn for safety-related reasons. One participant in the integrated CBT for ED-PTSD condition went to the emergency department due to suicidal ideation in the context of writing her trauma account but was not admitted to the hospital. She subsequently construed her suicidal ideation as a means of avoidance that was maintaining her PTSD and went on to complete treatment with no further safety-related events. There were no suicide attempts or psychiatric hospitalizations reported by participants.

**Table 1.** Participant characteristics

	CBT for ED (*N* = 23)	CBT for ED-PTSD (*N* = 19)	Overall (*N* = 42)
Gender (*n*, %)			
Woman	23 (100%)	18 (94.7%)	41 (97.6%)
Man	0 (0%)	1 (5.3%)	1 (2.4%)
Age (mean, s.d.)	29.7 (8.9)	28.5 (9.7)	29.2 (9.2)
Ethnicity (*n*, %)			
White	21 (91.3%)	15 (78.9%)	36 (85.7%)
Non-white	2 (8.7%)	4 (21.1%)	6 (14.3%)
Marital status (*n*, %)			
Single	14 (60.9%)	15 (78.9%)	29 (69.0%)
Married/Common-law	6 (26.0%)	3 (15.8%)	9 (21.5%)
Divorced/Separated	3 (13.0%)	1 (5.3%)	4 (9.5%)
Education (*n*, %)			
Less than high school	1 (4.3%)	0 (0%)	1 (2.4%)
High school/some college	13 (56.5%)	11 (57.9%)	24 (57.2%)
Undergraduate degree	9 (3.91%)	6 (31.6%)	15 (35.7%)
Graduate degree	0 (0%)	2 (10.5%)	2 (4.8%)
Financial situation (*n*, %)			
Fully/partially self-supported	13 (56.5%)	10 (52.6%)	23 (54.8%)
Dependent on others	10 (43.5%)	9 (47.4%)	19 (45.2%)
ED diagnosis (*n*, %)			
AN-R	1 (4.3%)	2 (10.5%)	3 (7.1%)
AN-BP	7 (30.4%)	3 (15.8%)	10 (23.8%)
BN	10 (43.5%)	10 (52.6%)	20 (47.6%)
OSFED	5 (21.7%)	4 (21.1%)	9 (21.4%)
Weeks in intensive treatment (mean, s.d.)	9.85 (3.11)	9.74 (3.63)	9.80 (3.31)
BMI (mean, s.d.)	20.74 (3.81)	22.78 (6.73)	21.66 (5.36)
Duration of ED in years (mean, s.d.)	12.71 (7.96)	11.42 (10.32)	12.13 (9.01)
Duration of PTSD in years (mean, s.d.)	8.28 (8.53)	11.31 (9.08)	9.65 (8.81)
Years since index trauma (mean, s.d.)	12.30 (11.62)	14.32 (12.55)	13.21 (11.95)
Index event type (*n*, %)			
Sexual assault/abuse	16 (69.6%)	16 (84.2%)	32 (76.2%)
Physical assault/abuse	4 (17.4%)	3 (15.8%)	7 (16.7%)
Other	3 (13.0%)	0 (0%)	3 (7.1%)
Psychotropic medication (*n*, %)			
Any	19 (82.6%)	16 (84.2%)	35 (83.3%)
Antidepressant	16 (69.6%)	14 (73.7%)	30 (71.4%)
Antipsychotic	6 (26.1%)	5 (26.3%)	9 (21.5%)
Antianxiety	9 (39.1%)	6 (31.6%)	15 (35.7%)

*Note.* Gender, age, ethnicity, marital status, education, financial situation, ED diagnosis, and duration of ED were assessed at the time of admission to intensive ED treatment, all other variables were assessed at study baseline (i.e. at the end of intensive ED treatment). ED, eating disorder; PTSD, posttraumatic stress disorder; CBT, cognitive behavioral therapy; AN-R, anorexia nervosa-restricting subtype; AN-BP, anorexia nervosa-binge-eating/purging subtype; BN, bulimia nervosa; OSFED, other specified feeding or eating disorder; BMI, body mass index.

### Mental health outcomes

There were no significant differences between conditions on the primary and secondary outcome measures at baseline. Table 2 shows estimated marginal mean scores on the outcome measures by therapy condition at each time point, as well as within-group effect sizes for baseline to EoT for each condition. Tests of fixed effects revealed significant interactions of condition and time on both clinician-rated and self-reported PTSD symptoms (CAPS-5 and PCL-5, respectively), *F*_(3, 45.26)_ = 4.06, *p* = 0.01, and *F*_(3, 41.65)_ = 7.13, *p* = 0.001, respectively. CAPS-5 scores decreased with both treatments, with a significantly greater decrease in the CBT for ED-PTSD condition than the CBT for ED only condition at EoT, 3-month, and 6-month follow-up. Pairwise comparisons indicated that improvements were maintained over follow-ups. The same pattern was found for PCL-5 scores. The condition by time interactions were not significant for anxiety, *F*_(3, 45.84)_ = 0.64, *p* = 0.60, depression, *F*_(3, 38.08)_ = 2.44, *p* = 0.08, or ED psychopathology, *F*_(3, 34.04)_ = 1.55, *p* = 0.22. However, estimates of fixed effects indicated that improvements in anxiety from baseline to 6-month follow-up were significantly greater in the CBT for ED-PTSD than the CBT for ED condition, *F*_(1, 19)_ = 5.48, *p* = 0.03. Between-group differences in anxiety were not significant for EoT and 3-month follow-up.

**Table 2.** Continuous outcomes at all-time points

	CBT for ED		CBT for ED-PTSD		Between groups effect size (*g*)
Time point	mean (95% CI)	Within effect size (*g*)	mean (95% CI)	Within effect size (*g*)	
CAPS-5					
Baseline	46.35 (42.34–50.35)	–	43.47 (39.07–47.88)	–	–
EoT	37.48 (31.61–43.35)	0.59*	24.10 (17.78–30.41)	1.27*	0.77*
3 mo FU	34.51 (28.68–40.33)	0.87*	21.54 (15.80–27.28)	1.55*	0.87*
6 mo FU	34.94 (27.17–42.71)	0.76*	20.73 (13.35–28.11)	1.44*	0.76*
PCL-5					
Baseline	58.42 (52.95–63.89)	–	53.74 (47.93–59.54)	–	–
EoT	49.40 (41.91–56.90)	0.49*	25.83 (18.02–33.64)	1.49*	1.15*
3 mo FU	46.44 (39.47–53.41)	0.70*	25.91 (19.07–32.75)	1.61*	1.19*
6 mo FU	47.79 (39.13–56.46)	0.61*	23.96 (16.12–31.80)	1.66*	1.27*
DASS anxiety					
Baseline	25.59 (21.48–29.69)	–	21.26 (16.91–25.62)	–	–
EoT	18.33 (13.70–22.95)	0.57*	12.04 (7.22–16.86)	0.72*	0.50
3 mo FU	20.63 (15.77–25.49)	0.40	12.29 (7.51–17.08)	0.71*	0.69*
6 mo FU	20.53 (15.10–25.95)	0.41	10.73 (5.80–15.66)	0.84*	0.83*
DASS depression					
Baseline	30.62 (26.50–34.74)	–	27.53 (23.16–31.90)	–	–
EoT	20.15 (13.74–26.55)	0.70	22.88 (16.22–29.55)	0.31	−0.16
3 mo FU	25.26 (18.96–31.55)	0.39	17.66 (11.50–23.83)	0.70	0.49
6 mo FU	24.93 (17.38–32.47)	0.41	22.71 (15.90–29.52)	0.33	0.14
EDE global					
Baseline	3.11 (2.44–3.78)	–	2.54 (1.81–3.27)	–	–
EoT	2.83 (2.23–3.43)	0.14	2.71 (2.07–3.36)	−0.09	0.06
3 mo FU	3.52 (2.87–4.18)	−0.20	2.88 (2.19–3.56)	−0.17	0.36
6 mo FU	3.47 (2.77–4.17)	−0.18	2.94 (2.23–3.66)	−0.20	0.30

*Note.* CAPS-5, Clinician-Administered PTSD Scale-5; PCL-5, PTSD Checklist-5; DASS, Depression Anxiety Stress Scales; EDE Global, Eating Disorder Examination 17.0 Global Score; ED, eating disorder; PTSD, posttraumatic stress disorder; EoT, end of treatment; mo, month; FU, follow up. *Denotes an effect size corresponding to a statistically significant pairwise comparison at the *p* < 0.05 level.

There were significant main effects of time on anxiety, depression, and ED psychopathology, *F*_(3, 45.84)_ = 12.36, *p* < 0.001; *F*_(3, 38.08)_ = 6.27, *p* = 0.001; and *F*_(3, 34.04)_ = 3.88, *p* = 0.02, respectively. Pairwise comparisons revealed DASS anxiety and depression scores improved significantly with both treatments, and that changes were maintained over follow-ups. There was also a significant main effect of condition on anxiety, *F*_(1, 37.44)_ = 8.11, *p* = 0.01. Across the two therapy conditions, pairwise comparisons revealed no significant changes in Global EDE scores over the course of treatment or follow-up.

Table 3 shows PTSD and ED diagnostic status at EoT and follow-ups. A greater proportion of participants no longer met the criteria for PTSD in the integrated CBT for ED-PTSD condition than in the CBT for ED condition at EoT and 3-month follow-up, Fisher's Exact, *p* = 0.002 and *p* = 0.03, respectively. The difference was not statistically significant at the 6-month follow-up. There were no significant differences between conditions in proportions of individuals who experienced reliable improvements and deteriorations in CAPS-5 scores over the course of treatment and at follow-ups. Overall, there were similar proportions of participants who experienced reliable improvements, deteriorations, and no change in EDE global scores between therapy conditions. There was no statistically significant difference in percentages of participants considered to no longer meet the diagnostic threshold for an ED.^†^^1^

**Table 3.** Reliable change and diagnostic status by treatment condition

	End-of-treatment		3-Month follow-up		6-Month follow-up	
Outcome	CBT ED *n* (%)	CBT ED-PTSD *n* (%)	CBT ED *n* (%)	CBT ED-PTSD *n* (%)	CBT ED *n* (%)	CBT ED-PTSD *n* (%)
PTSD (CAPS-5)						
Loss of diagnosis	1 (5.6)	9 (56.3)	3 (23.1)	10 (66.7)	3 (25.0)	8 (57.1)
Reliable improvement	5 (27.8)	9 (56.3)	6 (46.2)	12 (80.0)	6 (50.0)	9 (64.3)
Reliable deterioration	0 (0)	0 (0)	0 (0)	0 (0)	1 (8.3)	0 (0)
ED (EDE)						
Loss of diagnosis	12 (66.7)	10 (58.8)	11 (78.6)	7 (46.7)	9 (75.0)	8 (57.1)
Reliable improvement	3 (16.7)	1 (6.3)	2 (14.3)	1 (6.7)	2 (16.7)	2 (14.3)
Reliable deterioration	1 (5.6)	4 (25)	4 (28.6)	4 (26.7)	7 (58.3)	4 (28.6)

*Note.* PTSD, posttraumatic stress disorder; ED, eating disorder; CAPS-5, Clinician-Administered PTSD Scale-5; EDE, Eating Disorder Examination 17.0. Loss of PTSD diagnosis was defined as no longer meeting Diagnostic and Statistical Manual of Mental Disorders (Fifth Edition) symptom criteria as assessed with the CAPS-5; a reliable change represented a change from baseline of 11.64 points. Loss of ED diagnosis was defined as no more than 2 objective binge and/or purge episodes in the past month and a body mass index (BMI; weight in kilograms/height in meters^2^) of greater than 18.5; a reliable change represented a change in EDE global score of 0.91 points.

### Treatment satisfaction and preferences

Both treatments were rated highly with 96.8% at least *somewhat satisfied*, and 90.4% *very satisfied* to *completely satisfied*. There were no differences in treatment satisfaction between conditions, Mann–Whitney *U* = 0.40. All participants in both treatments who provided data said they would recommend the treatment they received to someone else. There was a strong preference for integrated treatment for both ED and PTSD (87.1%) over separate treatments for ED or PTSD. Finally, 93.3% of those who received integrated CBT thought having received PTSD treatment would facilitate their ED recovery.

## Discussion

This study represents the first controlled evaluation of integrated psychotherapy for ED-PTSD, which has been identified as a research priority in EDs (Obeid, McVey, Seale, Preskow, & Norris, 2020; Rijkers et al., 2019; Van Furth, van der Meer, & Cowan, 2016). We found strong evidence for the efficacy of integrated CBT for ED-PTSD in improving PTSD, in that the treatment led to large magnitude improvements in PTSD, and a higher rate of PTSD remission, relative to standard CBT for ED at EoT, with improvements maintained over 6-month follow-up. The lack of further improvement in ED psychopathology after intensive treatment that was found across both study conditions was likely related to the large magnitude improvements participants had already made during the intensive ED treatment that preceded the study treatments [pre-intensive treatment *M* = 4.40, s.d. = 1.12 and end-of-intensive treatment *M* = 2.85, s.d. = 1.09, *t* (41) = 9.95, *p* < 0.001, *g* = 1.01], and the likelihood of worsening ED symptomatology following intensive ED treatment even with additional treatment (Carter et al., 2008; MacDonald, McFarlane, Trottier, Mahan, & Olmsted, 2021). Ultimately, our hope is that integrated CBT for ED-PTSD will result in better ED outcomes than standard CBT for ED. However, this initial RCT was not powered to detect such differences, which would presumably be of a smaller magnitude in light of participants having already participated in intensive ED treatment. Indeed, the between-group effect size for ED psychopathology indicated a small to moderate magnitude benefit of integrated CBT over standard CBT for ED. Importantly, and in contrast to the expectations of some clinicians (Trottier et al., 2017), mixed models analyses and analyses of clinical status at EoT and follow-ups did not suggest worsening of ED or concomitant mental health symptoms in the integrated CBT for ED-PTSD condition relative to the standard CBT for ED condition. This is a vital first step toward demonstrating the safety and efficacy of integrating trauma-focused and ED psychotherapies. Results from this randomized efficacy-effectiveness study indicate that individuals with ED-PTSD can not only tolerate, but also greatly benefit from, manualized trauma-focused psychotherapy. This new information is critical to overcoming barriers to accessing evidence-based PTSD treatments related to clinician perceptions that trauma processing will lead to an increase in ED and other mental health symptoms, including PTSD symptoms (Trottier et al., 2017).

Although integrated CBT resulted in greater PTSD symptom reduction, higher PTSD remission rates, and larger magnitude improvements in anxiety, CBT for ED also led to improvements in PTSD, anxiety, and depression, as well as similar ED outcomes, and high treatment satisfaction. These findings raise questions about to whom integrated CBT should be provided. Given that integrated CBT for ED-PTSD led to superior PTSD outcomes, and that integrated treatment was highly preferred by participants and perceived by participants to facilitate their ED recovery, we suggest that integrated psychotherapy for ED-PTSD should be offered whenever possible. Further, given the high prevalence of co-occurring PTSD in those presenting for intensive ED treatment, the likelihood that PTSD and ED maintain one another when they co-occur, and high rates of relapse in EDs (Berends et al., 2018; Yu et al., 2013), we hope that these findings will spur a shift in practice such that evidence-based psychotherapy for PTSD is made available in ED treatment programs.

At the same time, we would be remiss to not highlight the good outcomes experienced in this study with standard CBT for ED. Psychiatric comorbidity is one of several reasons that clinicians tend to stray from providing manualized psychotherapy for EDs (Waller & Turner, 2016), and our findings indicate that in the case of ED-PTSD, manualized CBT for ED is beneficial. That said, the improvements in PTSD with standard CBT for ED may have been, in part, due to the assessment and treatment process being more trauma-informed than is typical of such treatments in research studies and clinical practice. The recruitment process, multiple assessments of PTSD symptoms, and the fact that the study therapists were experienced working with individuals who have PTSD, may have contributed to improvements in PTSD.

### Strengths and limitations

There are several important strengths of this initial trial, including that it was conducted in a clinical practice setting with program therapists, and that it utilized an active treatment comparison group, which was consistent with routine care. Several limitations should also be considered in interpreting and generalizing the results of this trial. Although adequately powered for our primary hypothesis, the study's ED diagnostic heterogeneity and relatively small sample size do not allow us to draw conclusions about the impact of integrated CBT for ED-PTSD on ED outcomes relative to standard CBT for ED. The sample size also limits examination of some factors that might moderate treatment outcome, such as type of trauma or duration of illness with PTSD or ED, as well as our ability to examine potential mediators of treatment response. Larger trials that can examine these questions, as well as whether similar results can be achieved at other sites are needed. Another potential limitation is that participants had to have completed a course of intensive ED treatment focused on nutritional stabilization and ED symptom interruption in order to be eligible to participate. Individuals who did not complete the course of intensive treatment and/or did not reach a BMI of 18.5 were selected out; thus, our sample may have been made up of individuals who are more likely to complete treatment. It is possible that non-completion rates may have been higher, and outcomes poorer, if participants had not already completed this course of treatment. That said, these inclusion criteria are consistent with recommendations in the literature regarding when to initiate trauma-focused treatment with individuals with EDs (e.g. Brewerton, 2004). The majority of participants were also taking psychotropic medication. It is possible that these medications may interact with the psychotherapies that were provided and outcomes may not generalize to those who are not on psychotropic medication.

Another important next step in developing and testing integrated psychotherapies for ED-PTSD is to adapt the protocol to be a stand-alone individual therapy (i.e. not requiring a course of intensive ED treatment beforehand). There are also many other important empirical questions to be answered such as at what minimum BMI trauma-focused psychotherapy can be effectively initiated in individuals with ED, whether engaging in ED behaviors such as binge eating and purging negatively affects outcomes of integrated treatment, and whether integration of other evidence-based CBTs for PTSD (e.g. prolonged exposure therapy; Foa, Hembree, Rothbaum, and Rauch, 2019) and EDs (e.g. integrative cognitive-affective therapy; Wonderlich et al., 2015) is also efficacious. We hope that these first RCT findings will catalyze change in the treatment of individuals with ED-PTSD and encourage further research on integrated psychotherapies with the goal of enabling more fulsome mental health recoveries for these individuals.

## Footnotes

† The notes appear after the main text.

## Note

1 Given the lack of a consensus definition, the impact of more and less stringent thresholds for loss of ED diagnosis were examined. As expected, the number of participants meeting the threshold increased with less stringent and decreased with more stringent definitions. The proportion of individuals meeting the threshold was similar in each treatment condition regardless of the threshold used.
