# Results of the First Randomized Controlled Trial of Integrated Cognitive-Behavioral Therapy for Eating Disorders and Posttraumatic Stress Disorder – CORRIGENDUM

**DOI:** 10.1017/S0033291721005365

**Published:** 2022-02

**Authors:** Kathryn Trottier, Candice M. Monson, Stephen A. Wonderlich, Ross D. Crosby

**Affiliations:** 1 Centre for Mental Health, University Health Network, Toronto, Canada; 2 Department of Psychiatry, University of Toronto, Toronto, Canada; 3 Department of Psychology, Ryerson University, Toronto, Canada; 4 Sanford Health, Fargo, North Dakota, USA; 5 Sanford Research, Fargo, North Dakota, USA; 6 Department of Psychiatry and Behavioral Science, University of North Dakota School of Medicine and Health Sciences, Grand Forks, North Dakota, USA

This article was published in Psychological Medicine with errors in the names of authors in a couple of references. This has now been updated and the correct names used.

The authors apologise for this error.
